# Classification of α-synuclein-induced changes in the AAV α-synuclein rat model of Parkinson’s disease using electrophysiological measurements of visual processing

**DOI:** 10.1038/s41598-020-68808-3

**Published:** 2020-07-17

**Authors:** Freja Gam Østergaard, Marc M. Himmelberg, Bettina Laursen, Hartwig R. Siebner, Alex R. Wade, Kenneth Vielsted Christensen

**Affiliations:** 1 Department of Translational Biology, H. Lundbeck A/S, Ottiliavej 9, 2500 Valby, Denmark; 2 Department of Psychology, The University of York, Heslington, York, YO10 5DD UK; 3 Danish Research Centre for Magnetic Resonance, Centre for Functional and Diagnostic Imaging and Research, Copenhagen University Hospital Hvidovre, Kettegård Alle 30, 2650 Hvidovre, Denmark; 4 Present Address: GELIFES, University of Groningen, Nijenborgh 7, 9747 AG Groningen, Netherlands; 5 Present Address: Institut de Recherches Servier – IDRS, 125 Chemin de Ronde, 78290 Croissy sur Seine, France

**Keywords:** Predictive markers, Visual system

## Abstract

Biomarkers suitable for early diagnosis and monitoring disease progression are the cornerstone of developing disease-modifying treatments for neurodegenerative diseases such as Parkinson’s disease (PD). Besides motor complications, PD is also characterized by deficits in visual processing. Here, we investigate how virally-mediated overexpression of α-synuclein in the *substantia nigra pars compacta* impacts visual processing in a well-established rodent model of PD. After a unilateral injection of vector, human α-synuclein was detected in the striatum and superior colliculus (SC). In parallel, there was a significant delay in the latency of the transient VEPs from the affected side of the SC in late stages of the disease. Inhibition of leucine-rich repeat kinase using PFE360 failed to rescue the VEP delay and instead *increased* the latency of the VEP waveform. A support vector machine classifier accurately classified rats according to their `disease state’ using frequency-domain data from steady-state visual evoked potentials (SSVEP). Overall, these findings indicate that the latency of the rodent VEP is sensitive to changes mediated by the increased expression of α-synuclein and especially when full overexpression is obtained, whereas the SSVEP facilitated detection of α-synuclein across reflects all stages of PD model progression.

## Introduction

Parkinson’s disease (PD) is a progressive neurodegenerative disorder affecting ~ 0.2 to 3.0% of the population. It is characterised by the core motor symptoms bradykinesia, resting tremor, rigidity, and postural instability^1,2^. PD is also characterized by secondary non-motor symptoms such as hyposmia, constipation, orthostatic hypotension, depression, sleep disorders, decline in cognitive abilities, and deficits in visual processing. Many of these are present already at early stages of PD, whereas classical motor-related symptoms are present in the middle stages^3,4^. The pathogenesis of PD develops during prodromal stages of the disease^5^. Consequently, there is a high unmet need for validation of new biomarkers that can be used to assess new disease-modifying drug treatments in early or prodromal stages of PD^6^.

According to the Braak staging hypothesis, PD is a progressive synucleinopathic disorder in which six disease stages are associated with a progressive pathology to neurological structures^7^. Pathological hallmarks of PD are the degeneration of dopaminergic neurons in the *substantia nigra pars compacta* (SNc)^8^ and Lewy bodies in addition to ‘Lewy neurites’: pathological inclusions of aggregated α-synuclein protein in neurons^9,10^. Duplications and triplications of the *SNCA* gene encoding the protein associated with autosomal dominant early onset PD are accompanied with post-mortem presentation of Lewy bodies^11^ which suggests that changes in the homeostasis of the α-synuclein protein, i.e. increasing expression levels 30–50% can impact risk and onset of disease. α-synuclein found in Lewy bodies has often undergone a posttranslational modification e.g. phosphorylation at Ser129^12^.

Changes in visual perception are common but often overlooked in PD. Some of the most consistent deficits are changes in contrast sensitivity^13,14^ and delays in visual evoked potentials (VEPs)^15,16^. The occurrence of these changes in PD have been linked to the spread of α-synuclein aggregates and to reductions of dopamine within the retinal amacrine cells^17,18^.

In the adeno-associated virus (AAV) α-synuclein rat model of PD, protein expression and structural pathology originates from the injection site in the SNc, spreading to the striatal and cortical regions^19,20^. In rodents, the SNc projects to the superior colliculus (SC)^21^, while indirect links exist between the SNc and visual cortex (VC)^22^. The SC is believed to be the most prominent retinal target in the rodent visual system, receiving ~ 90% of the projections from the retinal ganglion cells^23,24^. Thus, there is potential for changes in α-synuclein homeostasis to affect visual processing in a rodent model of PD. The function of the SC is less studied in humans, however, the projections from the SNc to the SC are believed to be involved in detecting salient visual events^21^.

Recent research reported changes in visual processing using *Drosophila* models of PD, identifying increased contrast sensitivity as a visual biomarker in young *Drosophila* expressing a gain-of-function variant of the human leucine-rich repeat kinase 2 (LRRK2-*G2019S*) causing autosomal dominant PD in humans^25^. In the fly, pharmacological inhibition of LRRK2-*G2019S* fully rescued visual perturbations. This research has expanded to include sensitive machine learning classification techniques that allow the investigation of subtle, but important differences in visual function^26,27^. Establishing the utility of machine learning techniques to identify new visual biomarkers in animal models of diseases is important, as these techniques can be used as a tool for testing new therapeutic drugs that aim to alter disease progression, and define cross-species biomarkers that could be translated to the human condition^28^.

Here, we investigated changes in visual processing caused by α-synuclein overexpression in a rodent model of PD, using electrophysiological measurements of VEP and steady-state visually evoked potentials (SSVEPs) recorded from the VC and SC. Previous work has established that overexpression of human α-synuclein in the SNc leads to changes in firing pattern in the subthalamic nucleus of the rat^19^. Importantly, genetic and pharmacological ablation of LRRK2 using PFE360 rescued α-synuclein mediated changes in firing^19^. From this, we hypothesise that the overexpression of α -synuclein and concomitant changes in protein homeostasis will cause changes in the VEP waveform, and such changes are likely to be rescued by LRRK2 inhibition. Previously, machine learning classifiers have proved successful in classifying *Drosophila* PD models into their correct group based on subtle differences in visual responses. Consequently, we aim to investigate whether we can similarly classify rodents into their correct group using SSVEP profiles in a sensitive support vector machine (SVM) classifier.

## Methods

All animal experimentation was carried out in accordance with the European Communities Council Directive (86/609/EEC), and in accordance with Danish law on care of laboratory animals. The protocols were approved by the Danish Animal Experiments Inspectorate (Forsøgsdyrstilsynet) prior to the initiation of the study.

### Animals and stereotaxic surgery

26 female Sprague–Dawley rats, weighing 225 g at arrival, were used in the study. They were housed in Makrolon type IV cages with wood bedding, a transparent shelter, nesting material and wooden sticks, in rooms with a temperature of 22 ± 1.5 °C and a humidity of 55–65%. Food and water was provided ad libitum. Rats were anesthetized using 2.0 ml/kg Hypnorm® in saline and midazolam (B.Braun, Melsungen, Germany) in a 2:1:1 relation (equivalent to fentanyl 157 µg/kg) and placed in a stereotaxic frame. Local anaesthetic (Marcain, 2.5 mg/ml bupivacaine, AstraZeneca, Albertslund, Denmark) was injected prior to incision. A small drill was used to make seven holes in total: above the SNc (AP: − 5.5, ML: + 2.0 DV: − 7.2) of the left hemisphere for injection of the AAV vector. Electrodes were implanted bilaterally in the SC (AP: − 6.0, ML: ± 1.0, DV: − 3.5) and VC (AP: − 6.0, ML: ± 4.0). The reference electrode was placed at (AT: + 8.0, ML: − 2.0) and the ground electrode at (AP: − 2.0, ML: + 4.0).

Rats were randomly assigned into one of two groups, α-synuclein or null, 13 rats in each group. The α-synuclein group received an injection of AAV vector into the left SNc, whereas the null group received an injection of the empty AAV vector. Both groups received injections of 3.0 μl of viral particles (30 × 10^10^ GC) carrying the rAAV2/5 viral vector (Vector biolabs, Malvern, PA, USA) expressing human wildtype *SNCA* (hSNCA) with a chimeric promoter containing part cytomegalovirus and a part of the synthetic chicken β-actin promoter. The injection was carried out using a 32G Hamilton cannula with an injection rate of 0.2 μl/min. The right hemisphere received no treatment. Electrodes with a 15 mm mounting screw (E363/20/2.4/S, PlasticsOne, VA, US) were inserted into the visual cortex, reference, and ground holes. Stranded electrodes (E363/3/Spc, PlasticsOne, VA, US) were placed in the SC. The electrode leads were gathered in a plastic pedestal (PlasticsOne, VA, US) as a chronic implant attached using dental cement RelyX™ Unicem (3 M, Denmark) and Fuji plus (GC, US).

The procedure for all rats was completed over 9 days. Norodyl (carprofen 5 mg/kg) (ScanVet, Fredensborg, Denmark) and Noromox prolongatum (amoxicillintrihydrat 150 mg/kg) (ScanVet, Fredensborg, Denmark) were administered during surgery and for five days post-surgery. Following surgery, the rats were kept on a reversed 12 h circadian cycle (lights on at 6:00 PM). Rats were left to recover and acclimate for 2 weeks.

### VEP and SSVEP stimuli

In the VEP experiment, rats were exposed to flashes of light presented at 1 Hz using five wavelength conditions to investigate interactions between α-synuclein overexpression and wavelength. The wavelengths were: red light (20 lx, 620–625 nm), green light (20 lx, 525–530 nm), blue light (20 lx, 455–460 nm), short-wave blue light (5 lx, 405 nm), and white light (20 lx, 400–700 nm). Each rat was presented with 400 repetitions of each wavelength condition of the VEP stimulus at each testing session. In the SSVEP experiments, rats were presented with flickering luminance stimuli that flickered with a continuous square wave at a frequency of 14 Hz, with each presentation run lasting 100 s. Again, the SSVEP stimulus had five wavelength conditions, identical to those used to evoke VEPs. The duration of each flash was 10 ms and there was no light in the cage between flashes. Lux was measured using an LED luxmeter (Extech, MA, US) located in the bottom of the cage. Stimuli were controlled by Spike2 ver. 7.13 (RRID:SCR_000903) (Cambridge Electronic Design Ltd, UK) and were presented using 5,050 SMD LEDs positioned 60 cm above the bottom of the cage.

### Recording of VEP and SSVEP

Rats were placed in a Makrolon type IV cage of polycarbonate with wood bedding and a Plexiglas box (measuring 43.8 × 35 × 27.9 cm) placed on top of the Makrolon cage. The test cage was placed inside a larger Faraday cage. Recordings were performed during the dark phase. VEP and SSVEP recordings were made at eight time points throughout an 11-week period (see Figure S1), counted from the first day of surgery at 3, 5, 6, 7, 8, 9, 10, and 11 weeks using Spike2 ver. 7.13 while the animals were awake and behaving. Electrophysiological signals were amplified and filtered using a Brownlee amplifier model 410 (Brownlee Precision, CA, US) at the following settings: low-pass filter; 200 Hz, high-pass filter; 1 Hz, sampling rate; 1,000 Hz.

### Cylinder test

Eleven weeks after the injection of AAV, the rats were tested for motor asymmetry induced by the injection of α-synuclein in the left SNc. This overexpression affects the striatum ipsilateral of the injection and reduces the use of the contralateral paws. Rats were placed in a transparent plastic cylinder, with one paw dyed red and the other dyed green. The animals were recorded for five minutes using a video camera. The number of touches on the plastic was counted and the ratio of contralateral to total number of touches was computed. The results from the two groups of rats were compared using an unpaired t-test.

### Administration of PFE360

PFE360 is a LRRK2 kinase inhibitor first described in Baptista et al.^29^. The drug was synthesized at Lundbeck A/S (Valby, Denmark) and dissolved in 10% captisol titrated to pH > 2 using 1 M methanesulphonic acid to a concentration of 3 mg/ml. The rats were administered perorally with a dosage corresponding to 7.5 mg/kg of PFE360. The dosing was carried out as a randomized cross-over study 14–15 weeks after the injection of the AAV vector. EEG recordings were initiated 1 h after administration at the expected maximum brain concentration (C_max_)^19^.

### Post-mortem assessment of α-synuclein overexpression

Brains were divided into three parts for western blotting, assessment of compound concentration in the brain, and histological validation of electrode location. The rostral part containing the striatum was frozen and used for western blotting. The medial part from 13 of the 26 rats (seven control animals and six from the α-synuclein group) was immersion fixed and the medial part from the remaining 13 rats (six control animals and seven from the α-synuclein group) were used for western blotting. The immersion fixed brain parts were used for immunohistochemistry (this is shown in Figure S6), as the medial region contains the SC. The cerebellum was used to determine the brain concentration of PFE360.

### Western blotting for α-synuclein, pS129-α-synuclein, striatal enriched phosphatase (STEP_46_), tyrosine hydroxylase (TH), gamma-aminobutyric acid transporter (GAT1), neuronal marker (NeuN), LRRK2 and LRRK2-pS935

The procedure for tissue preparation and western blotting is described in detail in Andersen et al.^19^. In brief, tissue punches were taken from the striatum and the SC and were homogenized using a Precellys® lysing kit (Bertin Instruments, France) with CelLytic M buffer (Merck, Germany) and proteases. Protein was isolated and the concentration was determined with BCA Protein Assay (Thermo Scientific, MA, US). For SDS-PAGE and western blotting procedures, 2.5 µg of protein was added in each well. Proteins were transferred unto immobilon-FL PVDF membranes (Millipore, Billerica, US). The membranes were incubated over night at 4 °C with primary antibody: human wildtype α-synuclein (1:10,000; 4B12; Thermo scientific, US), pSer129-α-synuclein (1:1,000; ab51253; RabMAbs®, Abcam,UK), STEP (1:1,000; 23E5; Millipore, US), TH (1:2000; ab152; Abcam, UK) , GAT1 (1:500; ab426; Abcam, UK), NeuN (1:1,000; ab104225; Abcam, UK), LRRK2 (1:2000; N241A/34; NeuroMab, US), LRRK2-pS935 (1:1,000; [udd2 10(12)] ab133450; Abcam, UK). The antibodies were detected using a Licor Odyssey system (LI-COR Biosciences, NE, US) with fluorescent secondary antibodies and a Chameleon™ duo pre-stained protein ladder (LI-COR Biosciences, NE, US). Intensity values for the fluorescence of the TH band were measured using the Licor Image Studio Ver. 3.1.4 (LI-COR Biosciences, NE, US). STEP_46_ immunoreactivity (IR) on Western Blot membranes was used as a dissection and loading control for tissue punched from the striatum. The STEP_46_ isoform encoded by the PTPN5 gene is highly enriched in cells of striatal origin as opposed to the STEP_61_ isoform^30^. In addition, by evaluating STEP_46_ IR a qualitative measure for striatal excitotoxity associated with the AAV model was also ensured^31^.

### Histology

To pilot the rationale of a VEP study in the AAV model of PD, immunohistochemistry was performed on four perfusion fixed brains from a previous study of the AAV-model in our lab. The brains were extracted at week 10–11 after injection, and cut in slices of 40 µm on a freeze microtome and placed in potassium phosphate buffered saline (KPBS). The tissue was quenched with hydrogen peroxide and washed in KPBS before being incubated with primary antibody hWT-α-synuclein (4B12) (Thermo scientific, US) in the concentration 1:1,000. Tissue was then washed before being incubated with the secondary antibody (E0464, DAKO, Denmark) and exposed using a 3,3-diaminobenzidine-reaction.

### Plasma and brain concentration of PFE360

The animals were decapitated after the final recording session, approximately 3 h after the last PFE360 dosing. Blood and brain tissue from the cerebellum were sampled after the final EEG recordings to determine the peripheral and central exposure of PFE360. The procedure for sampling and determination of the PFE360 concentration is described in detail in Andersen et al.^19^.

### VEP analysis

The peak amplitude and the latencies of the flash VEPs were extracted from the Spike2 waveform display. We defined the peak amplitude as the difference from baseline to peak, measured in millivolts (mV). The latency of the peaks was measured relative to the stimulus offset. The SSVEP data was exported from Spike2 and further analysed in MATLAB 2018a (RRID:SCR_001622) (Mathworks, MA, US).

The latency and amplitude of each peak of the VEP waveform were extracted manually in Spike2, for each rat and wavelength condition. Grand averages were computed for each electrode over all 400 stimulus repetitions. Statistical analysis for the amplitude and latency of the VEP, and the data obtained from the cylinder test, was carried out using the RStudio interface for R ver. 3.4.2 (RRID:SCR_000432) (Rstudio, MA, US).

Individual ANOVAs were conducted for both VC and SC. This resulted in 24 separate comparisons which were corrected for multiple comparisons using false discovery rate. As the AAV injection was unilateral, the contralateral hemisphere was considered an internal control. This means that an effect of AAV-mediated α-synuclein overexpression on visual processing would be apparent as an interaction between the treatment group and the side in which the recording was made. The three-way ANOVAs included the variables side, group, and colour. The principles of the backward variable elimination strategy was applied to assess whether any of the variables could be discarded^32^. Post-hoc comparisons were carried out using the Tukey multiple comparison test.

For the PFE360 experiments, a four-way ANOVA was applied with drug as an additional factor. The analysis was carried out using the backward variable elimination strategy, followed by Tukey post-hoc comparisons if *p* < 0.05.

### SSVEP analysis

SSVEPs are phase-locked electrophysiological signals that are traditionally analysed in the frequency domain, rather than the time domain^33^. SSVEP data were analysed separately at each week using MATLAB 2018a (RRID:SCR_001622) (Mathworks, MA, USA). The data was comprised of 100 1 s bins. Figure S 2A shows an example of an averaged EEG time course across 1 s. The first 5 s and the last 5 s of each SSVEP time course was excluded to account for onset transients and offset artefacts, thus retaining 90 1 s bins of EEG data per rat for each electrode and wavelength, at each week. These data were transformed into the power spectrum by running a fast Fourier transform (FFT) on each 1 s bin. As presented in Figure S2B, amplitude peaks occurred at multiples of our input frequency, namely 1*f* (14 Hz) and 2*f* (28 Hz). We retained the Fourier amplitudes from these two frequencies as our frequencies of interest.

To filter the data, the mean noise was computed by averaging the amplitudes in the four frequency bins above and below the two frequencies of interest (14 Hz and 28 Hz). To create a signal to noise (SNR) estimate, we compared the Fourier amplitude at 1*f* and 2*f* to the root mean square of the noise calculated from these local side bins. Any amplitude bins that had an SNR lower than 1 were excluded. Thus, all amplitudes retained for further analysis had a Fourier amplitude that was larger than the average noise across the neighbouring bins of 1*f* and 2*f* on the power spectrum.

The SNR measurements were used to minimize the variation contributed by noise in the SSVEP. We used a three-way ANOVA to test the α-synuclein induced differences in SSVEPs within week 11. The variables were side, group, and colour.

Phase data were analysed using the CircStat toolbox for Matlab^34^. A hktest (two-factor ANOVA, directional statistics)^35^ was computed for the pooled wavelength conditions. The independent variables were group and side.

### Machine learning classification and bootstrapping

The goal of using the SVM classifier was to accurately classify rats based on multiple features of the SSVEP amplitude drawn from different electrode configurations. Each rat had a total of 40 potential SSVEP amplitude features that could be used in the classification (i.e. amplitudes from 5 illumination wavelengths, 2 harmonics, and 4 electrodes) with 90 1 s bins of data for each feature. It is possible to apply a machine learning classifier to the two groups of rats and compute an overall classification accuracy. However, this can lead to issues of overfitting. To circumvent this, the classification procedure was bootstrapped to perform multiple classification iterations on groups of `synthetic’ rats by sampling data from the group-level population.

The classification estimates were bootstrapped by repeatedly sampling (without replacement) from the 1 s bin pool of Fourier amplitudes. For each of the four electrodes, this sampling pool contained a maximum of 1,170 Fourier amplitude bins (13 rats × 90 1 s bins). Wavelength was included as a feature, rather than classifying *into* wavelength, as an increase in features often benefits classification accuracy. We randomly permutated through these amplitude bins, assigning 90 random 1 s bins from each feature to each synthetic rat. These 90 1 s bins were then averaged together for each feature. The phase data was not included in the SVM, as the summation of phase response across individual 1 s SSVEP bins may yield inaccurate phase information.

This created 13 synthetic α-synuclein rats and 13 synthetic control rats. Amplitude responses were normalized by Z-scoring the data across each synthetic rat. The Z-score of each feature was entered into the SVM classifier and labelled with the class that the data corresponded to; α-synuclein or control rat. Unique variations of these synthetic rats were run through 1,000 bootstrapped iterations of the SVM classifier to derive a mean classification accuracy and corresponding significance value. The mean classification accuracy was deemed significant if less than 5% of the 1,000 iterations fell below a 50% chance baseline, equivalent to a *p*-value of 0.05. In an additional analysis, we shuffled our rat labels on each run. If the SVM is working correctly, we would expect the mean classification accuracy of shuffled data to fall around chance (50%).

### Support vector machine

The SVM is a robust supervised learning model based on statistical learning theory developed and first implemented by Vladimir Vapnik^36^. The SVM uses a non-linear hyperplane as classification boundary to assign new examples of data to either class, with the output being the accuracy of the SVM in classifying new examples of data^37–39^. We bootstrapped a SVM classification analysis in MATLAB 2018a using LIBSVM, ver. 3.23^40^. The SVM had a radial basis kernel function and a 5-sample k-fold cross validation, with four groups of data used as the training data and a single group used as the validation data for each run to avoid overfitting.

## Results

### AAV-mediated overexpression of α-synuclein

**Figure 1:**
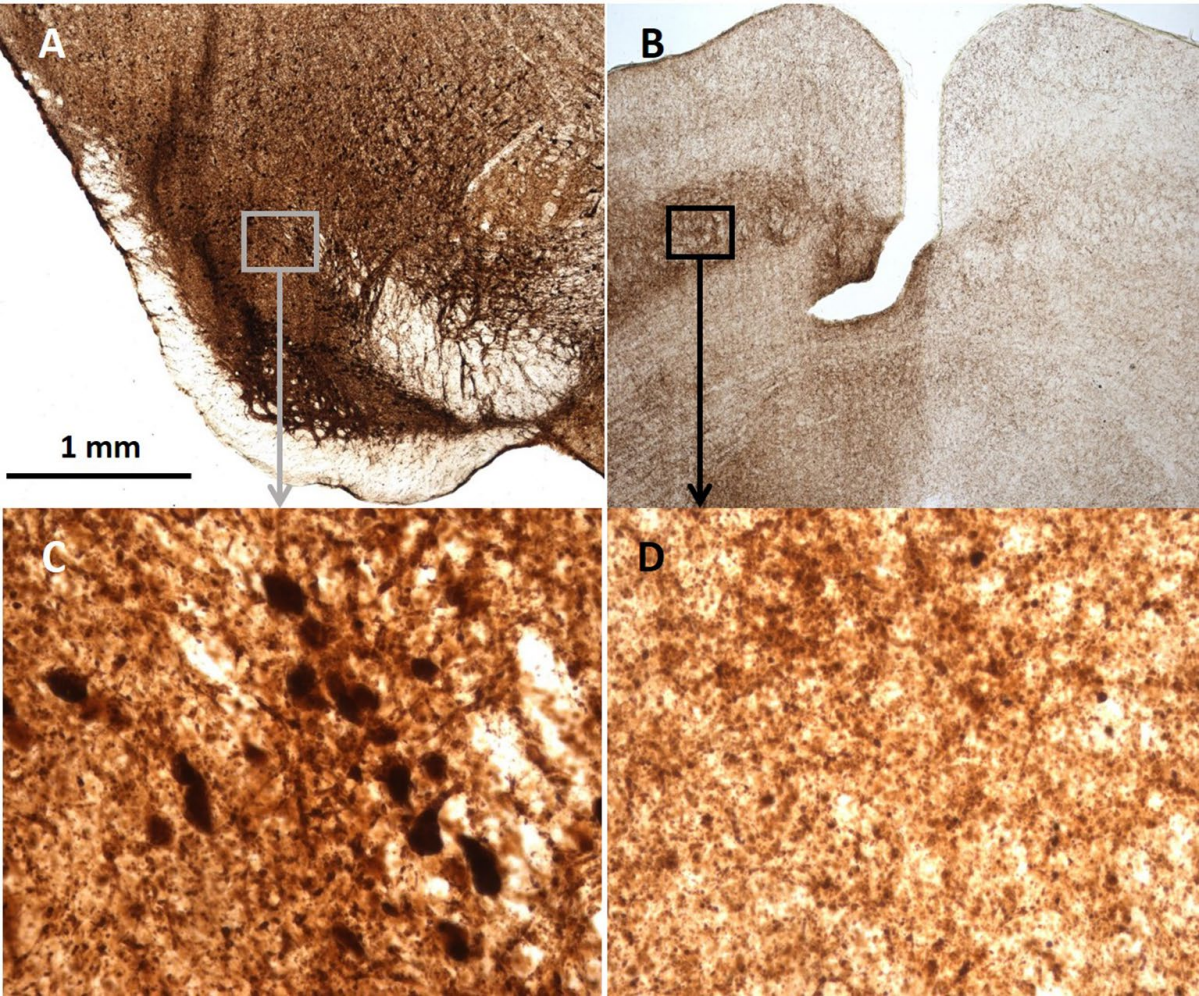
Detection of human α-synuclein immunoreactivity (IR) in the superior colliculus of rats injected with AAV- α-synuclein. (**A–D**) Images of immersion fixed brain slice stained with the 4B12 antibody for human wild-type α-synuclein and magnified 10 × and 40 × . (**A**) Human wt α-synuclein IR in the substantia nigra pars compacta (SNc). (**C**) A subsection of the human α-synuclein IR in the SNc at 40 × magnification. The magnification highlights cell bodies containing human wt α-synuclein. (**B**–**D**) Human wt α-synuclein IR appearing in the intermediate gray layer of the superior colliculus (SC).

To investigate the extent of spreading of the α-synuclein protein to the visual regions in the rats, the brains from rats injected with the AAV-h*SNCA* vector were collected at week 10–11 and stained for human wild-type α-synuclein (Fig. 1). All brains (n = 4) showed an abundance of α-synuclein expression in the ipsilateral SNc and surrounding areas, exclusively ipsilateral to the injection. Immunoreactivity towards human α-synuclein was also observed in the ipsilateral SC. No α-synuclein immunoreactivity was detectable in the contralateral hemisphere, although minor unspecific binding around the edges of the tissue was observed.

### α-synuclein is associated with a behavioural motor deficit in the AAV α-synuclein rat

The rats completed the cylinder test in week 11 to assess motor asymmetries associated with the behavioural consequences of α-synuclein overexpression. The cylinder test (Figure S3A) showed a significant motor asymmetry in the group overexpressing α-synuclein, but not in the control group. There was a statistical significant difference in the mean ratio of contralateral touches to total number of touches (t(21) =  − 3.99, *p* < 0.001). The mean ratio for the α-synuclein rats was 0.34 indicating that 34% of touches were with the paw contralateral to the side of injection (i.e. right paw). The control group used the two front paws equally, with a mean ratio of 0.49.

Further, as the immunohistochemistry was carried out on brains from a different study (see methods), western blotting was applied to validate the presence of exogenous human α-synuclein in the striatum and SC of the animals used in the present study. For a representative blot, see (Figure S3B and C). The western blots indicated that in the α-synuclein rats, α-synuclein was present in the SC and striatum ipsilateral to the injection. Contrary to this pSer129-α-synuclein was detected using WB with samples from the striatum of both the control and the α-synuclein rats, further pSer129-α-synuclein could only be detected around the injection site using immunohistochemistry (Figure S6). STEP46 was used as a loading control and TH was quantified in the striatum as ratio of contralateral to ipsilateral to the injection. There was no asymmetry induced by expressing human α-synuclein (t(7) =  − 1.57,* p* = *0.16*) (Figure S4).

### Measurements of VEP

#### Visual cortex is not impacted by AAV-mediated α-synuclein overexpression

**Figure 2:**
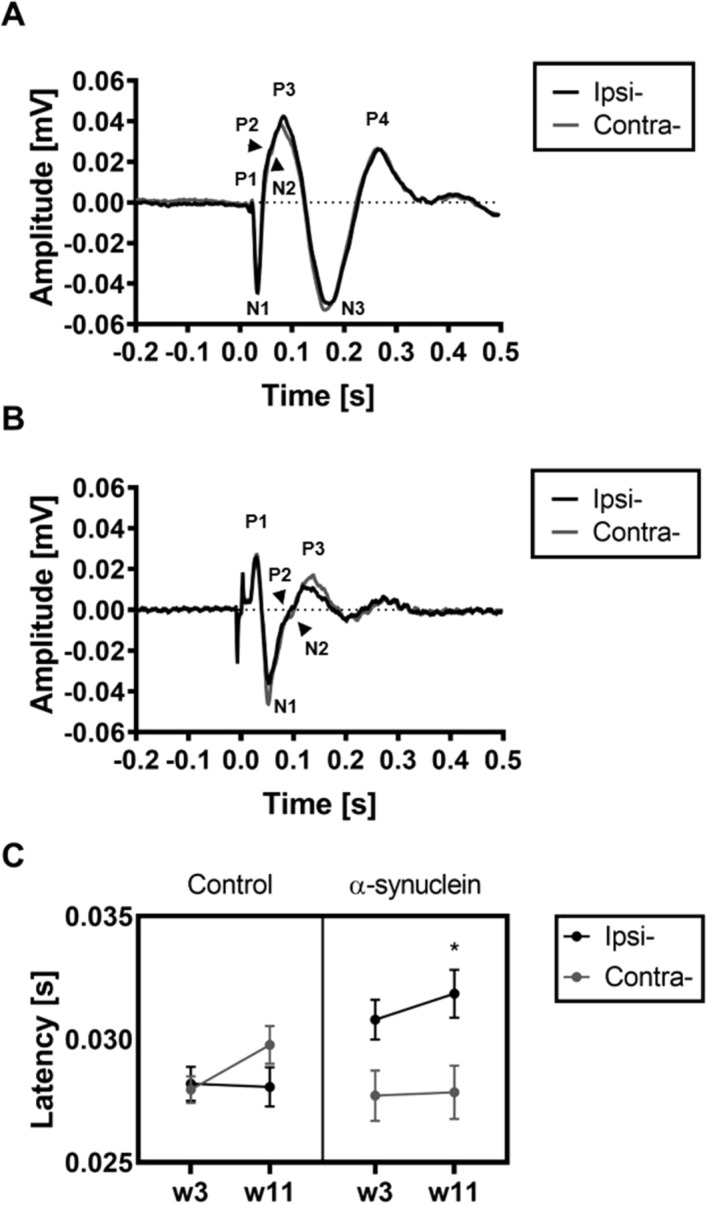
Unilateral α-synuclein overexpression in the SC causes a delay of the first positive peak (P1) of the VEP. (**A**) The grand average VEP waveforms from the visual cortex recorded ipsilateral (black) and contralateral (grey) to the injection of AAV-α-synuclein at week 11 (averaged across colour). (**B**) The grand average VEP waveform from the SC (averaged across colour) recorded from animals expressing α-synuclein in the left substantia nigra ipsilateral (black) and contralateral (grey) to the injection of AAV-α-synuclein at week 11 (averaged across colour). The peak around time 0 is an electrical artefact from the onset/offset of the VEP stimulus. (**C**) Latency of the P1 peak from the waveform recorded from the SC shown as mean ± SEM at week 3 and week 11 for both α-synuclein and control animals. The P1 recorded ipsilateral to the AAV-α-synuclein injection is significantly delayed at week 11 compared to P1 recorded contralateral to the injection. * *p* < 0.05.

Next, we investigated whether the overexpression of α-synuclein caused changes in the VEP measured from the VC. Figure 2A shows the grand average waveforms recorded from the right and the left VC in the AAV α-synuclein group at week 11. There was no significant difference in the amplitude or the latency of the VEP, when comparing between VC of the two hemispheres of α-synuclein and control rats. This finding is corroborated by histological findings where no evidence of α-synuclein inclusions were present in either hemisphere of the VC (data not shown).

#### α-synuclein alters VEP responses in the left SC

To investigate whether the overexpression of α-synuclein induced changes in visual processing we tested for differences between ipsi- and contralateral sides using the latency and amplitude data of the rodent VEP by running a three-way ANOVA using the data from week 11 (the final week of testing). The grand average of the waveform is shown in Fig. 2B. The wavelength variable was eliminated as it did not interact with the expression of α-synuclein. There was a significant effect of the interaction between side and group on the latency of P1 measured in the SC (*F*(1,24) = 12.32, *p* = 0.002). The post-hoc comparisons revealed a 0.004 s mean latency increase for the left (α-synuclein expressing) SC (0.004 s, SE = 0.0013, *p* = 0.012) when compared to the right SC in the α-synuclein group. Similarly, there was a significant increase in latency (0.003 s, SE: 0.0013, *p* = 0.019) when comparing left SC in the α-synuclein rats and the control rats. There was no significant difference in latency between the right SC of α-synuclein group and control group (− 0.0019 s, SE = 0.0013, *p* = 0.44). These findings suggest that the VEP obtained from the side of the injection in the α-synuclein animals is functionally affected, congruent with our histological inspections and results from the cylinder test.

Next, we investigated whether the difference in response latency was evident at the earliest stage in the model. Figure 2C shows that the difference in latency between the right and left hemisphere increases over time in the α-synuclein rats. This is not observed in the control rats. A three-way ANOVA on the effect of side group and colour did not show a significant interaction of side and group on the latency of P1 in week three (w3) (*F*(1,24) = 2.78, *p* = 0.11). This suggests that the increase in latency developed over time in parallel with the increase in expression of human α-synuclein. These results indicate that the VEP waveform is sensitive to the overexpression of α-synuclein at the later stages in the model, as evidenced by increases in the latency of P1 in the left SC of the α-synuclein injected rats.

#### Modulation of the VEP by the LRRK2 inhibitor PFE360

**Figure 3:**
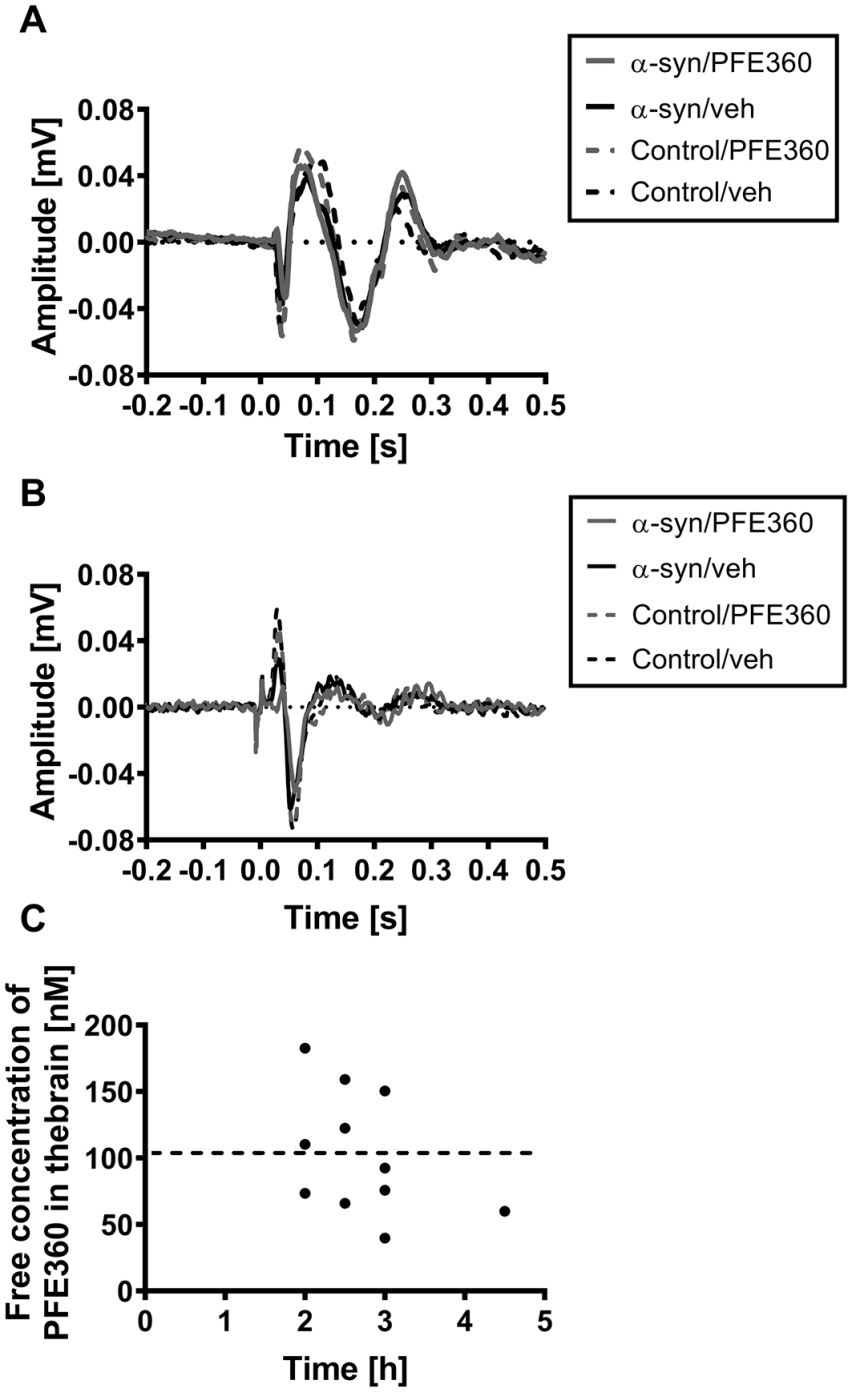
PFE360-mediated LRRK2 inhibition throughout the recording session. (**A**) shows four superimposed VEP waveforms recorded from the VC of rats injected with either AAV-α-synuclein (solid lines) or the empty AAV vector (dashed lines). Following this, they were treated with either vehicle (black) or the LRRK2 inhibitor PFE360 (grey). (**B**) Four superimposed VEP waveforms recorded from the SC. (**C**) Free concentration of PFE360 measured in the cerebellum of the rats, n = 11, 2–4.5 h after PFE360 administration. X-axis shows time since administration in hours. The broken line indicates the mean concentration.

In preclinical PD models, LRRK2 inhibition has been shown to normalize visual processing deficits in LRRK2-*G2019S* transgenic fruit flies^25^ and normalize increases in burst firing of the subthalamic nucleus in AAV α-synuclein rats^19^. Here, we investigate whether LRRK2 inhibition can rescue the phenotype caused by overexpression of α-synuclein at week 14–15 after injection. Figures 3A, B show the VEP waveforms (superimposed) of the grand averages from the four experimental groups: α-synuclein rats treated with either PFE360 or vehicle, and control rats treated with either PFE360 or vehicle. An increase in the latency of the waveforms was evident in both the SC and the VC in rats dosed with PFE360. Figure 3C shows the unbound concentration of PFE360 measured in the cerebellum after electrophysiological recordings. The mean unbound concentration of PFE360 was 103 ± 46 nM (mean ± SD, n = 11). One rat sacrificed 3 hr after acute administration of PFE360 had a free unbound brain concentration of 40 nM; significantly higher than the in vivo, IC50 of 2.3 nM (Andersen et al., 2018). This suggests that the LRRK2 kinase was inhibited throughout the recording sessions. Further, western blotting confirmed that, the inhibition caused a subsequent reduction of phosphorylated LRRK2-pSer935 (Fig. S6, in supplementary material).

**Figure 4:**
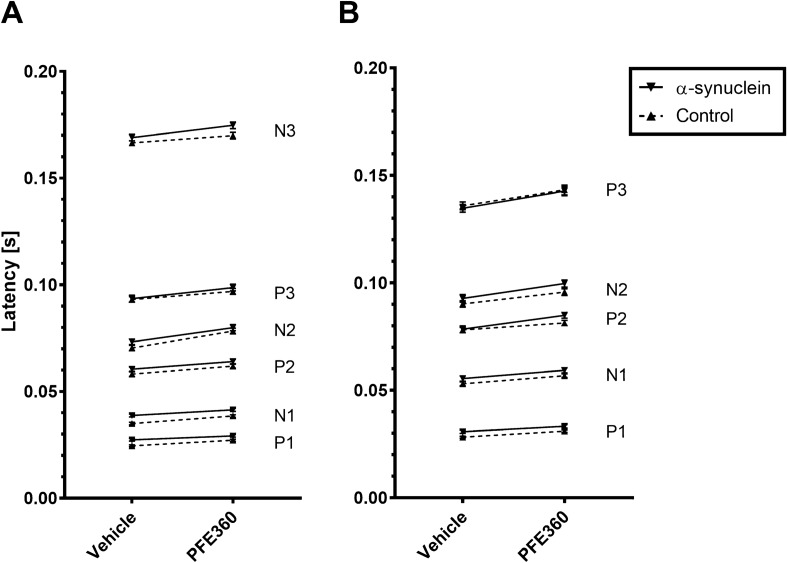
PFE360 causes an increase in the latency of VEP waveforms. All peaks are stacked to show the increase VEP latency. (**A**) Latency of each peak from the VEP recorded in the VC after administration of either vehicle or PFE360. There was significant increase in latency for all peaks except the P4 in both groups. The P4 peak from the VC was excluded as there was a statistically significant interaction of PFE360, wavelength, and group. (**B**) Latency of each peak from the VEP recorded from the SC after administration of either vehicle or PFE360. There was a statistically significant increase in latency for all peaks. Mean ± SEM, all differences between vehicle and PFE360 condition were significant with *p* values < 0.001 (see Table S1).

Running a 4-way ANOVA with the backward elimination of variables method^32^ replicated the interaction between group and side from the first study, showing a 3 ms increase in the latency of the P1 in the SC (Supplemental Table S1). Further, significant increases in mean latency of the N1 and N2 peaks of the VEP were observed with differences of mean of 6 ms, and 10 ms, respectively. There was no significant difference in the P2 and P3 peaks between the SC ipsi- and contralateral to the injection (data not shown). There was a significant three-way interaction of PFE360, wavelength, and group on the latency of P4 of the VC, and this peak was excluded from the visualization (Fig. 4). A significant additive effect of the PFE360 on the latency was observed for all other peaks in both the VC and the SC, but there was no detectable interaction between PFE360 and the α-synuclein overexpression (Supplemental Table S1). Finally, the latency of the peaks increased following PFE360 administration (Fig. 4). All peaks are shown in the same graph to illustrate the consistency of the observed effect. This suggests that a single acute dose of PFE360 has an impact on the rat visual response that is independent of any changes introduced by the overexpression of α-synuclein.

#### SSVEP shows an effect in the SNR of the first harmonic

Following the VEP findings, we tested for effects of the α-synuclein overexpression in the SSVEP data at week 11. The signal-to-noise ratio (SNR) of the amplitude data from the first harmonic of the VC showed a significant interaction of side and group (three-way ANOVA, *F*(1,244) = 6.84, *p* = 0.009), however post hoc comparisons found no significant differences. In the SC, there was a significant effect of side in the first harmonic (three-way ANOVA, *F*(1,244) = 4.60, *p* = 0.033), but no significant interaction of side and group. There were no significant differences for the second harmonic. The phase data from the SC showed no significant interaction of side and group in either harmonic and there was no significant difference in phase response from the VC when comparing between α-synuclein rats and control rats.

#### SVM can accurately classify α-synuclein rats vs. control rats

**Figure 5:**
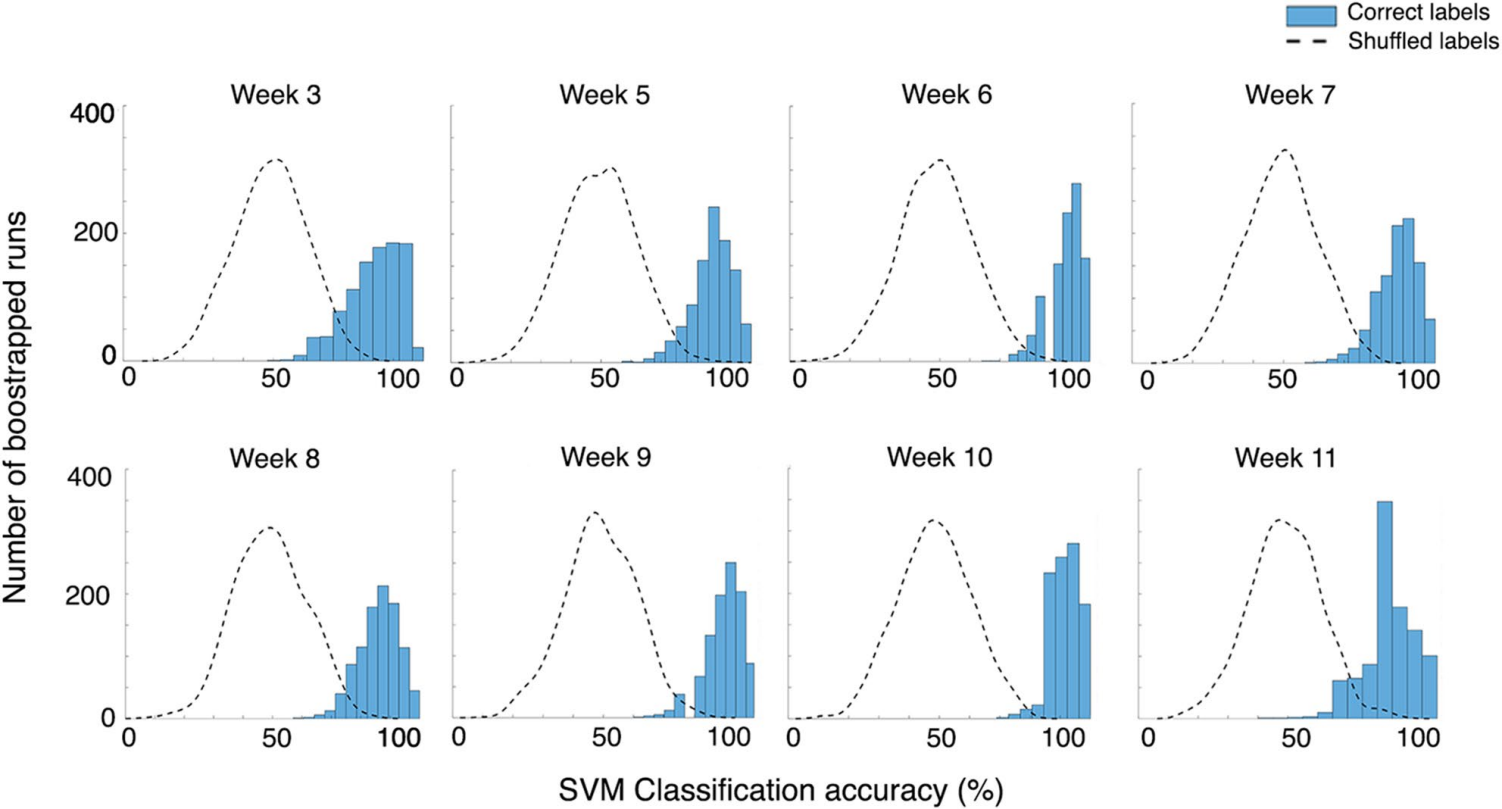
Histogram plots of classification accuracy across 1,000 bootstrapped SVM classifications. The SVM is highly accurate in classifying between α-synuclein and control rats at each week, when all electrodes are included in the analysis. Virtually no classification runs occurred below the 50% baseline. Plots of classification accuracy after shuffling labels are included (dotted lines). Here, accuracies fell around the 50% baseline.

Currently, the SVM is considered to be one of the most efficient methods of classification in real world applications. By applying the SVM we can find the hyperplane that best separates α-synuclein and control data. To test the ability of the SVM to accurately classify differences in visual processing, visual responses from the α-synuclein rat and control rat groups were compared, with amplitude data from all four electrodes included as features. The ability to distinguish between these two groups suggests significant variation in the SSVEP of these groups across the visual system. The average classification accuracy of 1,000 bootstrapped runs from each week and corresponding significance values are presented in (Table 1) and histograms of these bootstrapped accuracies are visualized (Fig. 5). The SVM was highly accurate in classifying rats into their respective groups at each week, with classifications consistently reaching > 83% accuracy. The SVM was tested with shuffled labels and found that average classification accuracies fell around 50% across all weeks (illustrated by the dotted line in Fig. 5). This was similar for both analyses, indicating that the SVM was working as expected. Shuffled label classification accuracies and *p* values are available in (Table S2).

**Table 1:** Mean SVM classification accuracy at each age after 1,000 bootstrapped runs, classifying α-synuclein rats and control rats into their correct class, when all electrodes were included in the analysis.

Week	Accuracy (%)	*p*-value
3	83.71**	*p* < .001
5	87.98**	*p* < .001
6	92.28**	*p* < .001
7	88.99**	*p* < .001
8	86.79**	*p* < .001
9	90.16**	*p* < .001
10	92.96**	*p* < .001
11	84.12*	*p* = .020

**p < 0.001, *p < 0.05.

#### SVM can accurately classify SSVEP responses from left and right SC

**Figure 6:**
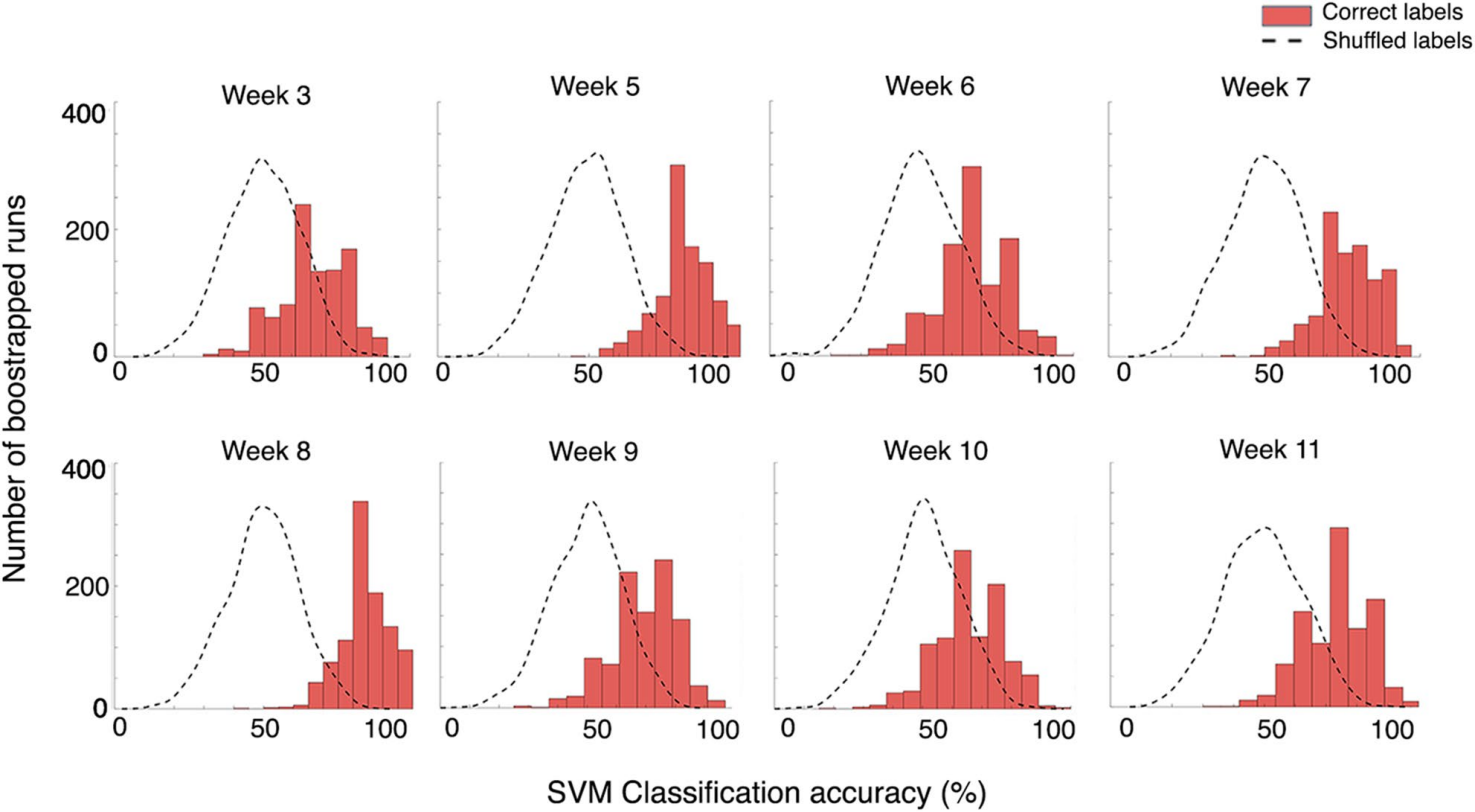
Histogram plots of classification accuracy across 1,000 bootstrapped SVM classifications. The SVM was able to classify between the (treated) left superior colliculus and the (untreated) right superior colliculus within α-synuclein rats, except at week 10. Plots of classification accuracy after shuffling labels are included. Here, accuracies fell around the 50% baseline.

Next, we assessed the ability of the SVM classifier in distinguishing between response amplitudes within the α-synuclein rats when only responses from the left (injected) and right (uninjected) SC were included in the analysis. The ability to distinguish between these two groups suggests significant variations in the SSVEP solely due to the expression of human wildtype α-synuclein. The average classification accuracy of 1,000 bootstrapped runs at each week and corresponding p-values are presented in (Table 2) and histograms of these bootstrapped accuracies (and shuffled accuracies) are visualized in (Fig. 6). Using SSVEP recordings from the α-synuclein group alone, the SVM was able to accurately classify between SSVEPs recorded from the left and right SC at all weeks, except at week 10, although this was closely approaching statistical significance. Notably, the SVM performed *better* when all electrodes were included in the analysis, rather than just the electrodes from the SC of the α-synuclein group.

**Table 2:** Mean SVM classification accuracy at each timepoint after 1,000 bootstrapped runs, comparing between the superior colliculus electrode ipsilateral to the injection and superior colliculus electrode contralateral to the injection within α-synuclein rats.

Week	Accuracy (%)	*p*-value
3	67.87*	*p* = 0.042
5	81.46**	*p* < 0.000
6	67.23*	*p* = 0.047
7	77.17*	*p* = 0.002
8	84.48*	*p* = 0.000
9	69.52*	*p* = 0.036
10	66.35	*p* = 0.062
11	73.01*	*p* = 0.032

**p < 0.001, *p < 0.05.

To sum up, these findings suggest that the SVM can accurately classify SSVEP responses in both α-synuclein rats and control rats across different electrode configurations. These results differ from our VEP results due to the highly sensitive nature of the SVM that can pick up nuanced differences in visual response profiles. Overall, it appears that the SSVEP is sensitive to the presence of the PD-associated gene product from early α-synuclein expression at week 3, through to the fully developed overexpression at week 11.

## Discussion

The purpose of this study was to investigate the potential of using VEP and SSVEP responses to detect changes in α-synuclein homeostasis in a rodent model of PD. First, in the α-synuclein rats, α-synuclein and pSer129 immunoreactivity was localized around the site of injection (the left SNc). Second, overexpression of α-synuclein was accompanied by an increase in the latency of the VEP measured from the ipsilateral SC, after α-synuclein expression was fully developed. Third, inhibition of LRRK2 using PFE360 could not rescue the increase in latency, but instead caused general increases in the latency of the VEP waveform. Fourth, the SVM was able to accurately classify rats according to their presence or absence of α-synuclein overexpression across time using two different electrode configurations.

### The overexpression of α-synuclein causes increases in the response latency of the VEP in the left SC, and this is congruent with the accumulation of α-synuclein

We asked whether induced overexpression of α-synuclein caused a detectable electrophysiological change in the rodent visual system as measured via the VEP and SSVEP. We have demonstrated that a small, but significant increase in the latency of the VEP measured from the left SC of α-synuclein rats occurs 11 weeks after injection. The result of our cylinder test suggests that the full impact of overexpressing α-synuclein is obtained 11 weeks after injection of rAAV2/5 similar to was has been reported by^19,41^. Thus, changes in the VEP waveform may only appear after full α-synuclein expression is obtained. This difference in VEP waveform was also present at 15 weeks where it seems to have developed further. This suggests that the functional consequences of overexpressing α-synuclein develop slower than as evidenced by the histological phenotype and might be further aggravated by downstream effects such as changes in α-synuclein homeostasis, phosphorylation or aggregation. The AAV-mediated expression of human α-synuclein was massively present in the entire *substantia nigra* both *pars compacta* and *pars reticulata.* In the rodent, the substantia nigra par reticulata (SNr) has direct projections to the SC^22^. This connection may explain how α-synuclein immunoreactivity are detectable in the SC, as the protein is transported and expressed within the cells. An alternative possibility being that the observed α-synuclein in the SC originates from the GABAergic processes connecting the SC and the SNc^42^. However, the exact mechanism by which α-synuclein increases the latency of the rodent VEP remains to be elucidated. Similar connections are found in humans, and so this could provide α-synuclein aggregates with a route to the SNr and SNc. In humans, α-synuclein have not been found in GABAergic cells^43^, whereas, aggregates of α-synuclein have been identified in the SC^44^.

### Treating α-synuclein rats with PFE360 caused a general delay in the VEP

The rodent AAV α-synuclein model may be used as a preclinical model to monitor disease-related progression and modulation by pharmacotherapy. Previous studies have found an interaction between LRRK2 function and α-synuclein induced phenotypes^19,45^. Similar to Andersen et al.^19^, we tested whether an acute dose of the LRRK2 kinase inhibitor PFE360 could modulate the VEP waveform. PFE360 caused an increase in the VEP latency which was observed for both the control and AAV-α-synuclein groups; however, treatment with PFE360 did not show any interaction with α-synuclein. This result does not exclude that α-synuclein and LRRK2 interact under other conditions or in other brain areas. PFE360 increases the general latencies of the VEP, suggesting a function for LRRK2 in facilitating post-synaptic potentials. Studies of the rodent brain have localised LRRK2 in the cortex and the striatum^46^ but not in the SC. Yet the present study indicates comparable effects in both the SC and the VC.

### The SSVEP showed changes in amplitude

There were general trends for changes in SSVEPs in α-synuclein rats, however post-hoc tests revealed that these trends were not significant. The SSVEP waveform originates from the summation of many repeated waveforms and while the temporal sensitivity and SNR are theoretically very high, it is possible that subtle differences in response waveform shape (such as we find in the event-related VEP) are obscured in the steady-state at the level of individual electrodes.

### SVM can accurately classify rats into their correct group using SSVEP profiles

Previously, it has been demonstrated that machine learning classifiers are a useful tool for establishing new visual biomarkers in *Drosophila* PD models^26,27^. Here, using information from all electrodes simultaneously, our SVM could accurately classify α-synuclein and control rats into their correct group across differing stages of α-synuclein overexpression. Rats could be classified into their correct group at all weeks with very high accuracy (84–93%). Second, SSVEP amplitudes from the left and right SC of α-synuclein rats were analysed using the SVM. The differences between these two groups were purely due to the injection of the AAV carrying human α-synuclein and not the empty vector AAV. We were able to classify rats into their correct group at most weeks—with significant accuracies between 67 and 84%.

One might expect the highest accuracy in the second analysis, comparing SSVEPs measured from the left SC (where α-synuclein inclusions were localized) with responses from the right SC that did not receive the AAV-vector and appeared structurally healthy. However, the SVM generally performed better when data from *all* electrodes were included in the classifier. One reason for this may be that SVM accuracy increases with increased number of features; in the first analysis 40 features were included, while the second analysis only included 10 features. Accuracy increasing with more features suggests that these additional features were relevant to the classifier itself, even though these data originated from electrodes where the effect of α-synuclein overexpression was not observable by VEP or immunohistochemistry. This indicates nuanced but significant differences in the SSVEP amplitudes that occur as a result of the overexpression of α-synuclein. Notably, the classifier identified differences in SSVEP response as early as three weeks after the administration of the AAV vector and these differences persisted across the progression of the increases of protein expression over time. Previous research has identified cell-to-cell transmission and the formation of exogenous α-synuclein fibrils in animal models expressing mutated *SNCA*^47^. Although we did not detect any human α-synuclein in the hemisphere contralateral to the injection site, our data suggest that the functional effect of α-synuclein overexpression may cause changes in long-range neural signalling that results in perturbations of the SSVEP measured from the opposing hemisphere.

There are clear benefits in combining electrophysiological measurements of visual processes and novel classification techniques. First, it may enhance early-stage drug discovery using animal disease models. Second, the accuracy (or inaccuracy) of a classifier in differentiating between visual processes recorded from a PD model that has been treated with a therapeutic drug against a healthy control animal allows for the objective assessment of whether a treatment has rescued visual functioning (i.e. are the responses between these two classes now indistinguishable). Further, with evidence for visual biomarkers in a range of simple fly models of PD, and now a more complex rodent model, it may soon be possible to translate such methods and classify human PD patients into their correct genotype based on similar electrophysiological response profiles^48^.

## Conclusion

We have established that the latency of the VEP is sensitive to overexpression of α-synuclein. This effect was localized to the site of α-synuclein overexpression. Next, we found that the administration of the LRRK2 inhibitor PFE360 caused general delays in the VEP which may suggest a function for LRRK2 in facilitating post-synaptic potentials. These delays were independent of the presence of human α-synuclein. Finally, we found that the SSVEP was sensitive to the presence of α-synuclein across multiple stages of α-synuclein overexpression and that such overexpression may cause changes in long-range neural signalling. These findings highlight the utility of the visual system to investigate changes in neural signalling that occurs in PD models. Overall, these findings may prove beneficial for producing methods that assist in the early diagnosis of PD in humans and testing new therapeutic treatments in disease models that aim to rescue neural responses.

## Supplementary information

Supplementary information.

## Data Availability

The extracted EEG data, can be provided upon request. The raw scans of western blot membranes can be provided upon request.
