# Supplementary material for: Classification of α-synuclein-induced changes in the AAV α-synuclein rat model of Parkinson’s disease using electrophysiological measurements of visual processing
Source: Sci Rep. 2020 Jul 17;10:11869. doi: 10.1038/s41598-020-68808-3 (PMC7368019; doi:10.1038/s41598-020-68808-3)
Supplement: Supplementary file 1 — Supplementary information. [file 41598_2020_68808_MOESM1_ESM.pdf]

Supplementary Material to 'Classification of α-synuclein-induced changes in the AAV α-synuclein rat model of Parkinson's disease using electrophysiological measurements of visual processing'

Freja Gam Østergaard[1*], Marc M. Himmelberg[2], Bettina Laursen[1], Hartwig R. Siebner[3], Alex R. Wade[2], and Kenneth Vielsted Christensen[1]

[1]Department of Translational Biology, H. Lundbeck A/S, Ottiliavej 9, DK-2500 Valby, Denmark. [2] Department of Psychology, The University of York, Heslington, York, YO10 5DD, United Kingdom. [3]Danish Research Centre for Magnetic Resonance, Centre for Functional and Diagnostic Imaging and Research, Copenhagen University Hospital Hvidovre, Kettegård Alle 30, DK-2650 Hvidovre, Denmark

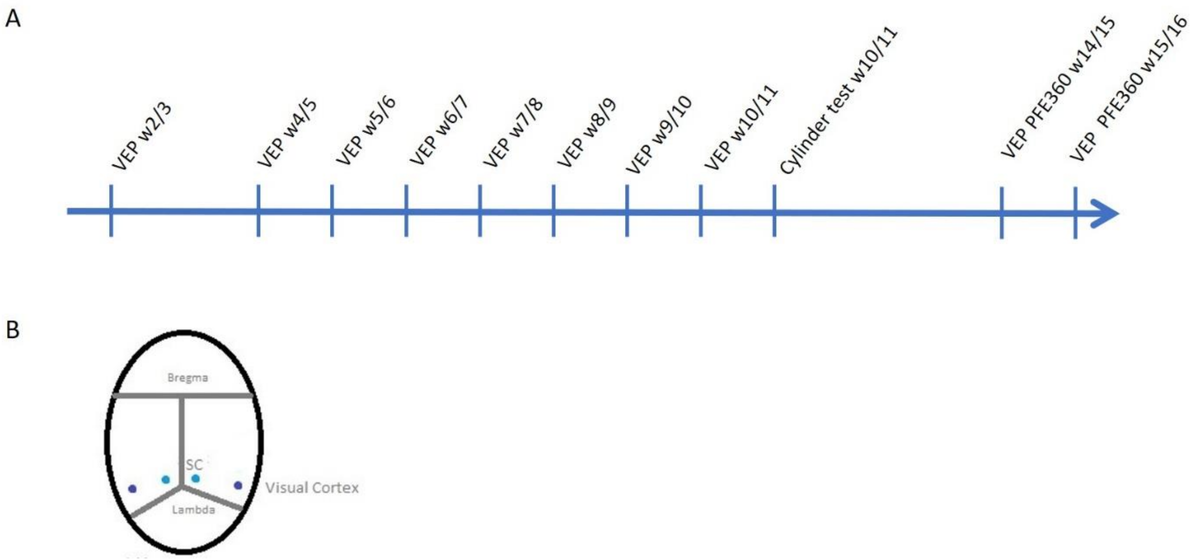

Figure S1 Sequence of electrophysiological recordings and behavioral tasks following intracranial implantation of recording electrodes. Rats recovered for 2 weeks after the surgery. **A)** Each rat completed ten SSVEP and VEP recording sessions (weeks 2/3 – weeks 10/11), the weeks are abbreviated w with a number giving the number of weeks since surgery. Then, rats completed the cylinder test in week 10/11 and PFE360 dosing and testing occurred in week 15. Rats were euthanized at week 16. **B)** Diagram of rat skull seen from above showing placement of the recording electrodes. The top of the figure is the anterior part towards the snout, the bottom part is the posterior part. Light blue spots near the midline delineates the positioning of the depth electrodes. Lateral dark blue spots show the positioning of the surface (screw) electrodes.

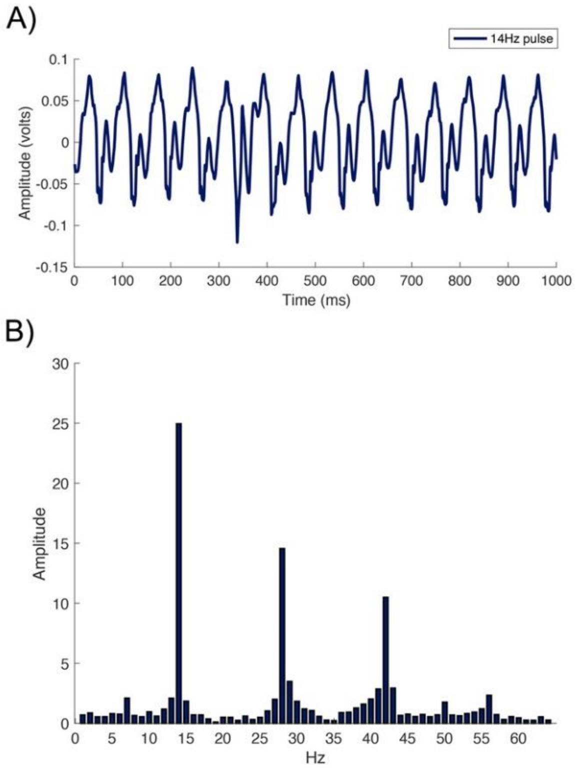

Figure S2 EEG data were collected in the time domain then transformed into the frequency domain using the FFT. In **A)** Example of the average EEG time course across a 1000ms bin taken from the right superior colliculus of a control rat three weeks after surgery. There are 14 peaks across the 1000ms bin, reflecting the 14Hz temporal frequency of our stimulus. In panel **B)**, we present the data in the power spectrum after applying the FT. Peaks occur at multiples of our input harmonic – at 14Hz, 28Hz, and 42Hz (1f, 2f, and 3f, respectively).

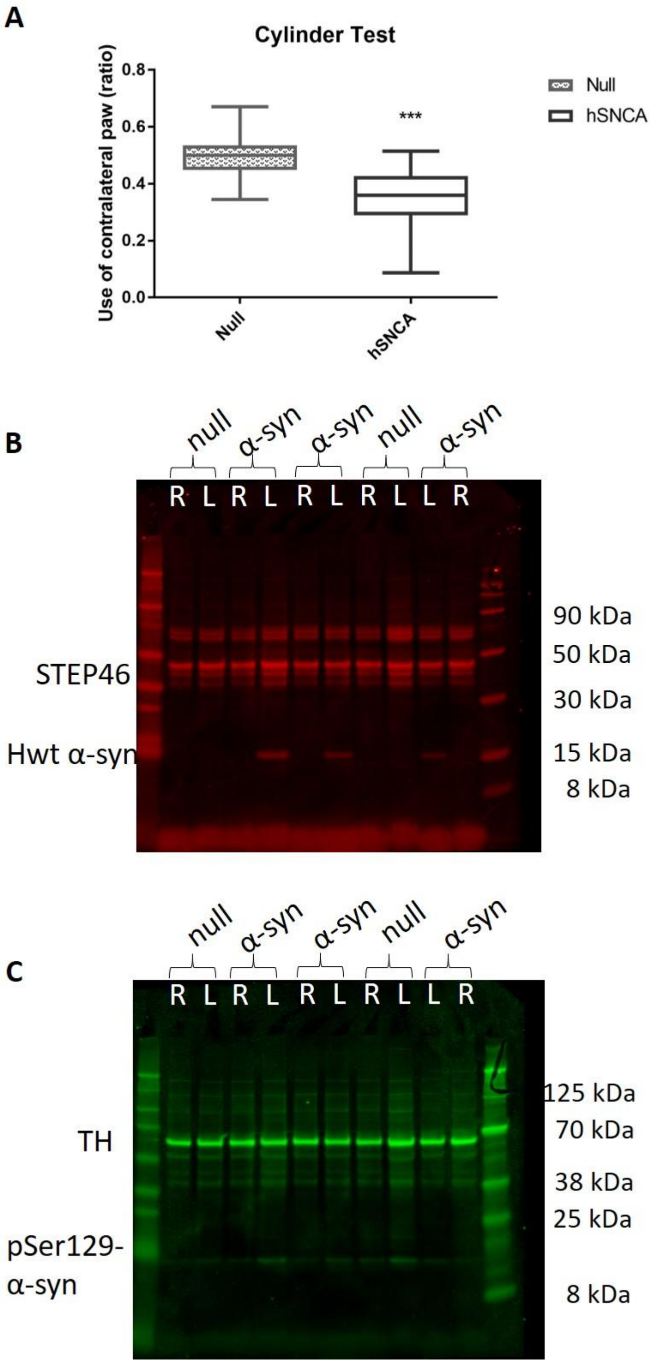

Figure S3 α -synuclein animals use the contralateral paw less than the ipsilateral paw. **A)** Ratio of contralateral touches over the total number of touches for the animals that had α -synuclein expressing AAV injected in the left SNc (black) and the animals that had the empty vector injected (grey). The null animals had ratios around 0.5, indicating that they use both paws equally, whereas the α-synuclein animals had ratios around 0.34 biased towards using the paw contralateral of the injection less, due to effects in the striatum. **B)** Representative western blot showing immunoreactivity (IR) of human α -synuclein, pSer129-α-synuclein, STEP 46 and tyrosine hydroxylase (TH) in protein lysates from the striatum. Human α-synuclein IR was only observed in samples from the ipsilateral hemisphere, whereas TH and STEP46 IR was detected in

both hemispheres. **C)** green filter applied to the membrane in **B)** with this setting TH and pSer129-α-synuclein becomes visible.

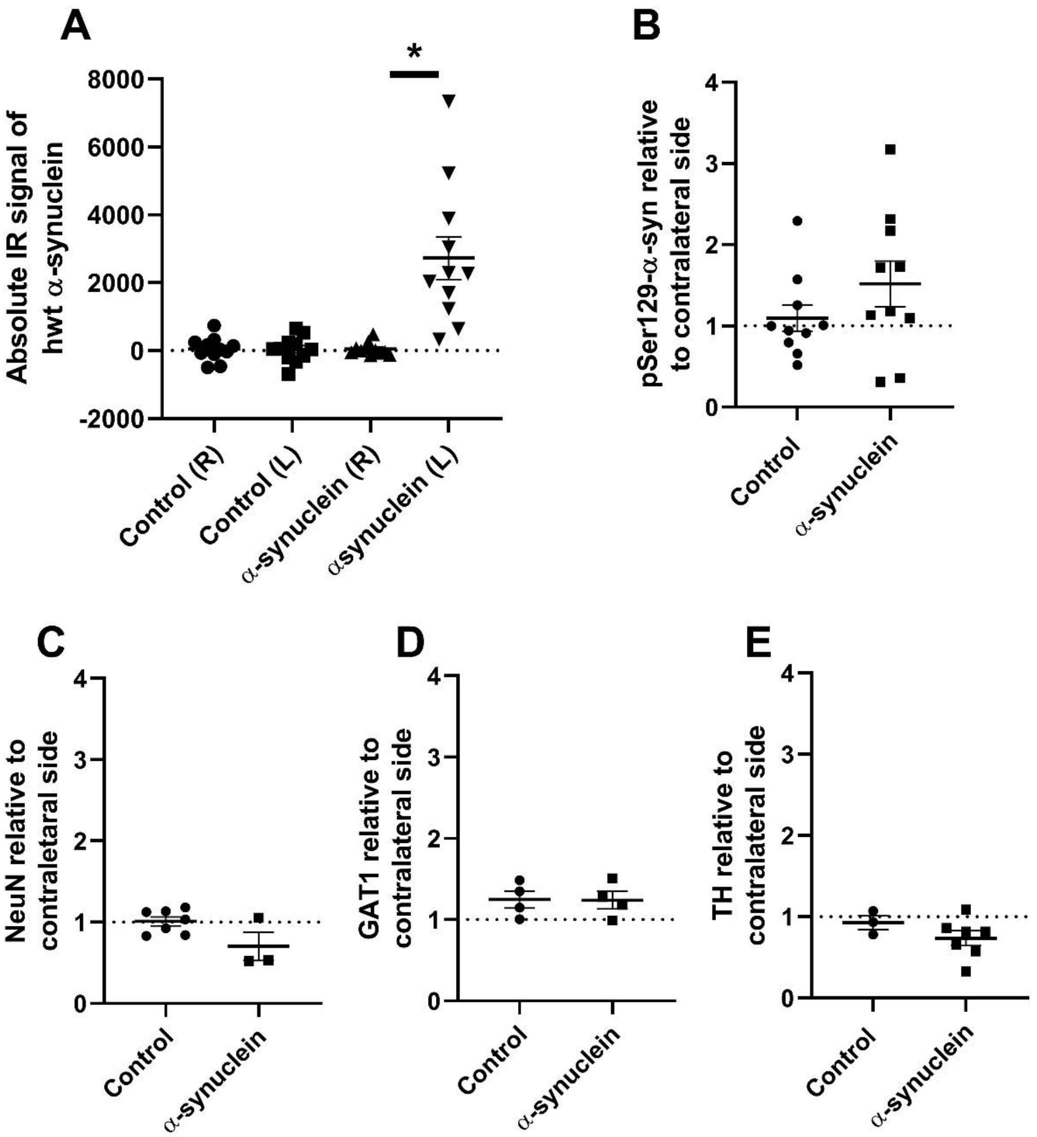

Figure S4 Western blot data from the striatum control and α-synuclein animals. **A)** Absolut values of infrared (IR) signal from both hemispheres of control and α-synuclein animals. Right (R) is contralateral to the injection with AAV vector. Left (L) is ipsilateral to the injection. Comparing R and L of the control yielded t(19.9)=0.051, p=0.96. Comparing R and L of the α-synuclein animals resulted in t(10.2)=-4.26, p=0.00161. **B)** the IR signal for pSer129-α-synuclein has been standardized to STEP46, and then a ratio of left to right has been computed. An unpaired t-test with inequal variance showed t(14)=-1.566, p=0.16. **C)** the IR signal for NeuN standardized to STEP46. **D)** the IR signal of GAT1 standardized to STEP, an unpaired t-test with inequal variance showed t(6)=-0.0517, p=0.961 . **E)** the IR signal of TH standardized to STEP. An unpaired t-test with inequal variance showed t(6.55)=-1.57, p=0.164.

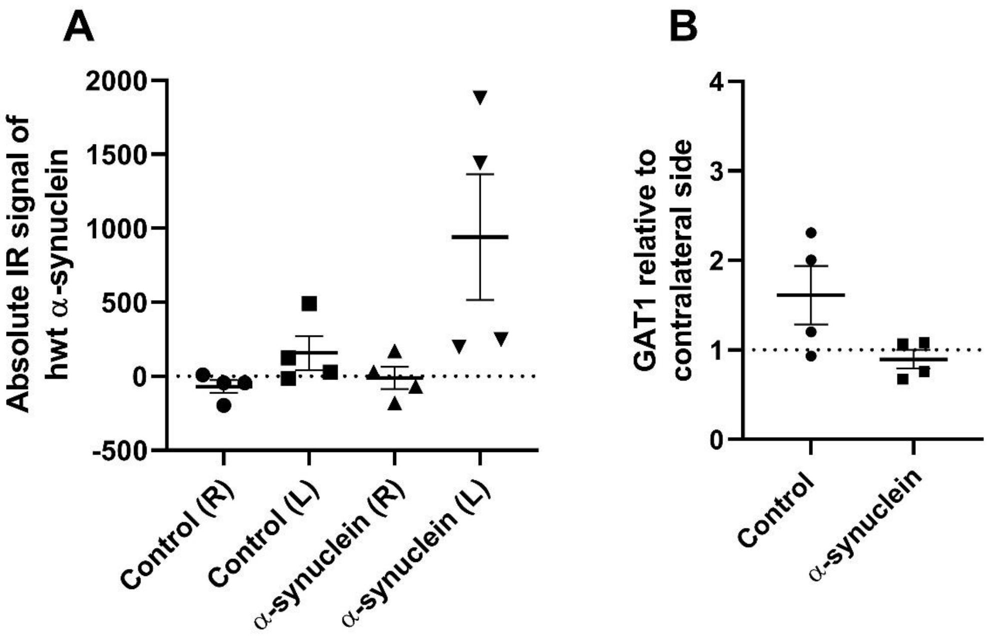

Figure S5 Western blot data from the superior colliculus of control and α-synuclein animals. **A)** Absolute values of infrared (IR) signal from both hemispheres of control and α-synuclein animals. Right (R) is contralateral to the injection with AAV vector. Left (L) is ipsilateral to the injection. Comparing R and L of the control yielded t(3.86)=-1.83, p=0.143. Comparing R and L of the α-synuclein animals resulted in t(3.19)=-2.21, p=0.109. **B)** the IR signal for GAT1 as the ratio left divided by right side. An unpaired t-test showed t(3.62)=-2.09, p=0.11.

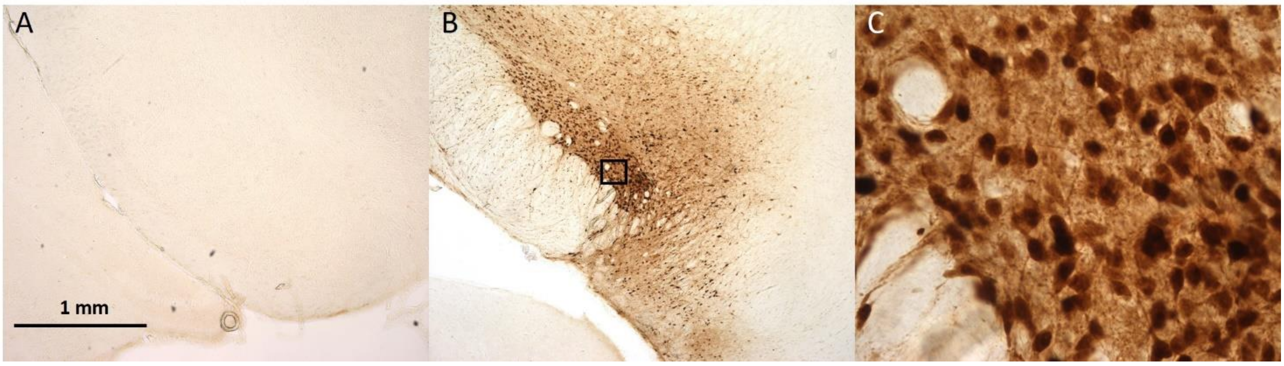

Figure S6 Detection of pSer129-α-synuclein immunoreactivity (IR) in the substantia nigra of rats injected with AAV- α-synuclein**. A-C)** Images of immersion fixed brain slices stained for pSer129-α-synuclein. **A)** Substantia nigra in the left hemisphere of a rat injected with the empty viral vector. **B)** Substantia nigra in the left hemisphere of a rat injected with a viral vector containing hwt α-synuclein. **C)** The black square in **B)** magnified 10 times.

Table of F-values (adjusted p-values) from 4-way ANOVA								2nd order interactions					3rd order interaction
								group			drug		
				group	side	drug	color	side	drug	color	side	color	group:drug:color
				numDF									
				1	1	1	4	1	1	4	1	4	4
Superior Colliculus	amplitude	P1	denDF 456		7.78 (0.0363)		5.21 (0.0033)	27.35 (0.00132)			trend		
		N1	471		13.18 (0.00293)								
		P2	456				8.84 (0.00132)	18.73 (0.00132)					
		N2	456				8.45 (0.00132)	13.23 (0.00293)					
		P3	456		14.27 (0.00189)		5.82 (0.00189)	31.43 (0.00132)	7.58 (0.0363)				
	latency	P1	455		27.91 (0.000471)	60.39 (0.000471)	145.32 (0.000471)	36.28 (0.000471)		4.68 (0.00495)			
		N1	455		8.95 (0.0106)	32.87 (0.000471)	57.32 (0.000471)	44.67 (0.000471)					
		P2	455			21.84 (0.000471)	31.96 (0.000471)	10.96 (0.00484)					
		N2	455			21.22 (0.000471)	19.35 (0.000471)	15.16 (0.000471)				trend	
		P3	455			19.55 (0.000471)	8.36 (0.000471)	10.40 (0.00582)					
Visual Cortex	amplitude	P1	464				4.34 (0.0145)	24.54 (0.000825)					
		N1	479				30.16 (0.000825)						
		P2	479			19.40 (0.000825)	27.55 (0.000825)						
		N2	479				22.30 (0.000825)						
		P3	464				5.41 (0.002933)	8.91 (0.0204)					
		N3	479				7.78 (0.000825)						
		P4	464		trend	trend	9.55 (0.000825)	18.16 (0.000825)				trend	
	latency	P1	479			57.67 (0.000471)	128.43 (0.000471)						
		N1	464	trend		81.14 (0.000471)	196.55 (0.000471)	trend					
		P2	479			28.18 (0.000471)	85.60 (0.000471)						
		N2	479		trend	51.58 (0.000471)	44.69 (0.000471)						
		P3	479			19.80 (0.000471)	34.02 (0.000471)						
		N3	464			16.32 (0.000471)	19.11 (0.000471)	11.89 (0.00308)					
		P4	451	trend			26.49 (0.000471)			4.11 (0.0113)		8.63 (0.000471)	4.46 (0.0066)

Table S1 Results of 4-way ANOVA, with F-values and FDR adjusted p-values in parenthesis. Fourth order interactions were not significant. Only significant interaction (after p-correction) are included in the table. numDF: numerator degrees of freedom, denDF: denominator degrees of freedom.

Week	Accuracy	***p*-value**
3	49.11%	p = 0.455
5	49.39%	p = 0.462
6	48.82%	p = 0.476
7	49.14%	p = 0.457
8	49.35%	p = 0.462
9	49.05%	p = 0.479
10	48.54%	p = 0.498
11	49.04%	p = 0.471

Table S2 Mean SVM classification accuracy at each week after 1000 bootstrapped runs, classifying α-synuclein rats and control rats into their correct class after labels have been shuffled, when all electrodes were included in the analysis. ** $p < 0.001$, * $p < 0.05$.

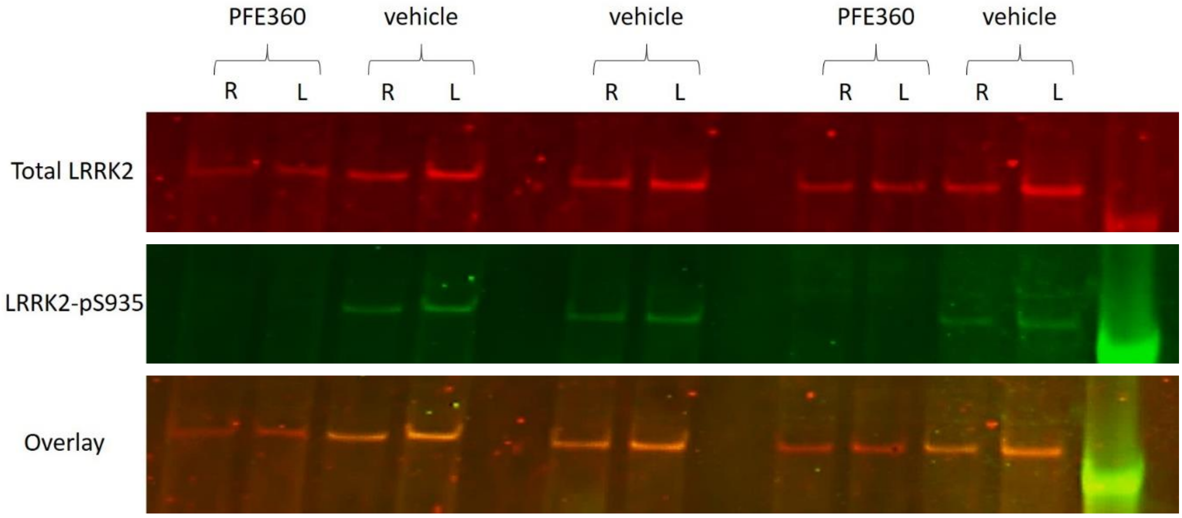

Figure S7 Expression and phosphorylation of LRRK2 in the rat striatum after administering PFE360. The three panels show the same part of the same gel, using three different filter settings. The top panel shows western blot detection of the total LRRK2 IR in protein lysates from the right and left side of the striatum, respectively. The middle panel shows the signal for phosphorylated LRRK2 (pSer935) in the same tissue samples. The bottom panel shows the IR-signals when superimposed. The results confirm that LRRK2 is fully inhibited in animals dosed with PFE360.

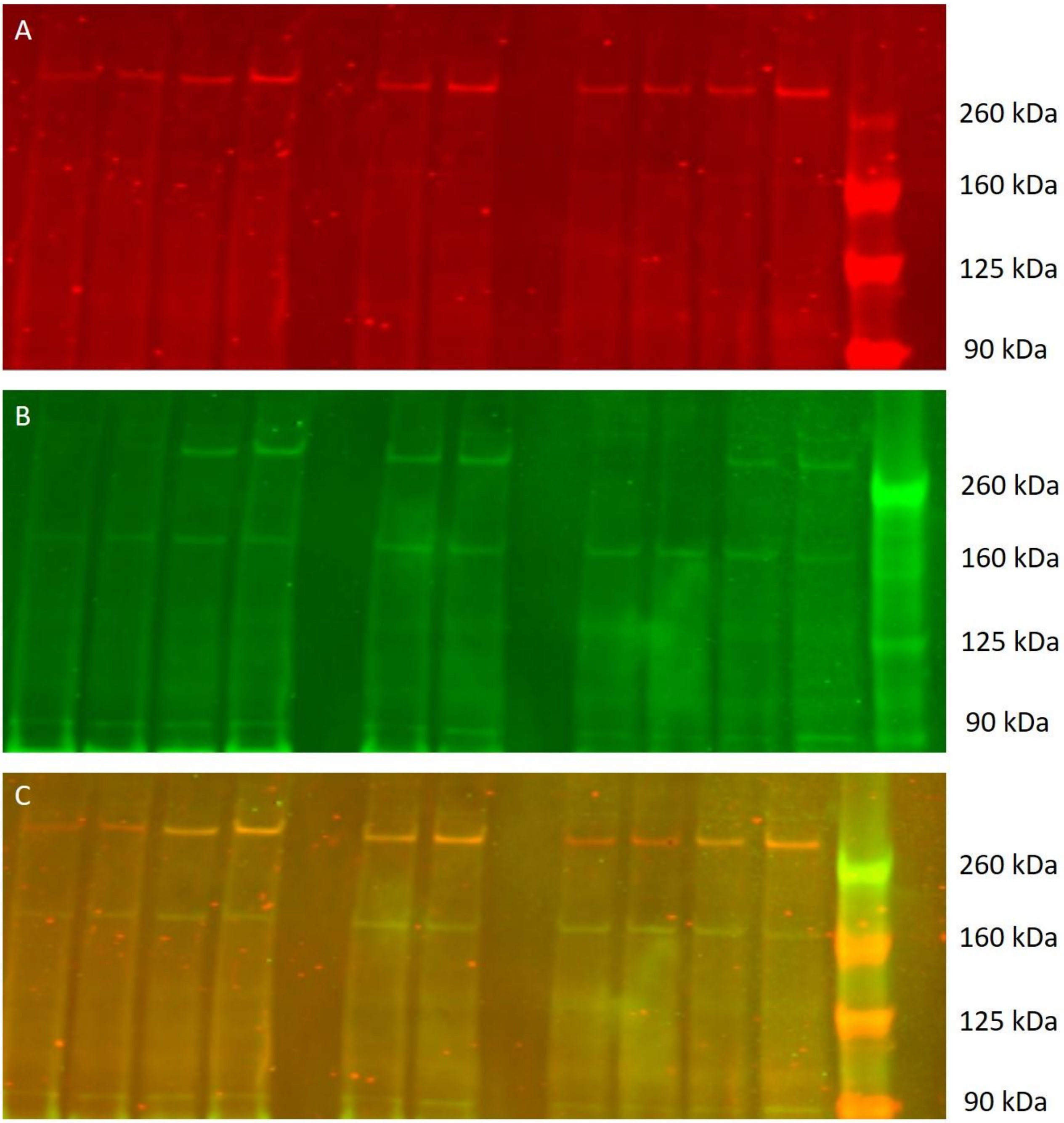

S8 Uncropped western blots used in figure **S7** with three different filters. **A)** red filter **B)** green filter **C)** both filters. The contrast settings are the same in all three panels.
